# Chlamydia psittaci in ocular adnexa MALT lymphoma: a possible role in lymphomagenesis and a different geographical distribution

**DOI:** 10.1186/1750-9378-7-8

**Published:** 2012-04-02

**Authors:** Francesca Collina, Anna De Chiara, Amalia De Renzo, Gaetano De Rosa, Gerardo Botti, Renato Franco

**Affiliations:** 1 Pathology Unit, National Cancer Institute "Giovanni Pascale", Naples, Italy; 2 Hematology Institute, "Federico II" University, Naples, Italy; 3 Biomorphological and Functional Sciences Department, "Federico II" University, Naples, Italy; 4 Pathology Unit, National Cancer Institute "Giovanni Pascale", Via Mariano Semmola, 80131 Naples, Italy

**Keywords:** Chlamydia psittaci, Ocular Adnexa, MALT lymphoma, t(14;18), bcl10

## Abstract

Ocular adnexa MALT-lymphomas represent approximatively 5-15% of all extranodal lymphomas. Almost 75% of OAMLs are localized in orbital fat, while 25% of cases involves conjunctive. MALT-lymphomas often recognize specific environmental factors responsible of lymphoma development and progression. In particular as Helicobacter pylori in gastric MALT lymphomas, other bacterial infections have been recognized related to MALT lymphomas in specific site. Recently Chlamydia psittaci has been identified in Ocular Adnexa MALT lymphomas, with variable frequence dependently from geographic areas. Thus bacterial infection is responsible of clonal selection on induced MALT with subsequent lymphoma development. Moreover Chlamydia psittaci could promote chromosomal aberration either through genetic instability as a consequence of induced proliferation and probably through DNA oxidative damage. The most common translocation described in MALT lymphomas affects NF-kB pathway with a substantial antiapoptotic effect. Several therapeutic approaches are now available, but the use of antibiotic-therapy in specific cases, although with conflicting results, could improve the treatment of ocular adnexa MALT lymphomas. In this review we analyse the most relevant features of Ocular adnexa MALT lymphomas, underlining specific biological characteristics mainly related to the potential role of Chlamydia psittaci in lymphomagenesis.

## Background

Non-Hodgkin's lymphomas represent the most common ocular adnexa neoplasm [1]. Approximately accounting 5-15% of all extranodal lymphomas localize at ocular adnexa, including the conjunctiva, the lachrymal gland, the orbital fat, the eyelid and the lachrymal sac [2]. In Western countries Marginal zone B-cell lymphoma of mucosa-associated lymphoid tissue (MALT)-type accounts 50-78% of all ocular lymphomas, while in Japan and Korea the relative frequence is much higher [3]. It has been recorded that Ocular Adnexal MALT Lymphoma (OAML) increases by more than 6% for year. These data are only partially explained by the development of recent new lymphoma classification and improvement of diagnostic approaches [4].

MALT-lymphomas represent a wide range of extranodal lymphomas that often recognize environmental factors driving to specific genes deregulation. Interestingly, environmental factors are specifically related to sites of acquired MALT development and furthermore the deregulated genes, even if involved in the NF-kB pathway, share different distribution in extranodal sites [5,6]. Among environmental factors, some bacterial infections have been found associated to MALT lymphomas of specific anatomic districts, such as H. pylori in gastric MALT lymphoma, C. Jejuni in intestinal MALT lymphoma and Borrelia Burgdoferi in cutaneous B cell lymphoma [6-10]. Recently Chlamydia psittaci (Cp) has been identified in OAML [11-25]. Chromosomal aberrations involving mainly MALT1 and bcl10 genes with consequent deregulation of their gene expression are commonly observed in MALT lymphomas, but with different distribution dependently from specific anatomic regions, being t(11;18) more frequent in gastric MALT lymphomas and t (14;18) in extragastric MALT lymphoma [26-29]. Moreover environmental and biological features show a relevant variability also related to geographic area. In fact Cp frequence in OAML has been described with difference frequence all over the world (Table 1) [11-25].

**Table 1:** Worldwide distribution of Cp infection

Geographical area	% Cp+	Reference
**North Italy**	87%	[11]

**Korea**	79%	[13]

**Austria**	54%	[14]

**Germany**	47%	[15]

**East coast USA**	35%	[15]

**Netherlands**	29%-0%	[15] &[24]

**Central Italy**	13%-16%	[15] &[17]

**UK**	12%	[15]

**Southern China**	11%	[15]

**Cuba**	10%	[16]

**Africa**	0%	[17]

**Rochester (New York)**	0%	[18]

**Southern Florida**	0%	[19] &[21]

**North China**	0%	[20]

**Japan**	0%	[22] &[23]

Thus this variability reflects different therapeutic strategies, that apart conventional chemotherapy and radiotherapy, are also based on specific targets or biomarkers useful to predict therapeutic response [30].

## Clinical features

Almost 75% of OAMLs are localized in orbital fat, while 25% of cases involves conjunctive [19,31-34]. Bilateral involvement has been described in 15% of OAMLs [19,31-34]. It arises after fourth decade, more frequently in female [19,31-34]. 38% of patients show at least one extraorbital site with lymphoma localization at accurate staging of OAML [35] In some cases autoimmune disorders are concomitant [36].

Clinical presentation depends upon involved orbital region, being exophthalmos (27% of cases), palpable mass (19%), eyelid ptosis (6%), diplopia (2%), eyelid nodule, orbital edema, epiphora and a variable degree of impaired ocular motility the most frequent signs and symptoms for orbital fat lymphomas, while 'salmon red patch' is the most common feature of conjunctival lymphomas [18,30-33]. Rarely impairment of extraocular muscle and infiltration of eyes have been recorded [37]. Diagnosis requires integrated study through computed tomography, magnetic resonance and imaging (MRI) and A- and B-scan orbital ultrasonography.

OAML patients exhibit a favorable outcome, when adequately treated [19,38]. Nodal involvement (< 5% of cases), systemic symptoms (1%), increased lactate dehydrogenase serum levels (1%) and non-conjunctival sites are considered negative prognostic factors [18,31,32]. Moreover these parameters seem to be related to high-grade transformation, recorded in 1-3% of cases [38,39]. 5-year relapse-free survival rate is 65%. Systemic dissemination has been observed in 5-10% of cases and only 5% of OAML patients die of lymphoma, with a 5-year overall survival of > 90% [33,34]. Some cases of spontaneous tumor remission in OAML patients have been reported, mostly in Japanese patients with conjunctival MALT lymphoma [40].

## Histopathology

**Figure 1:**
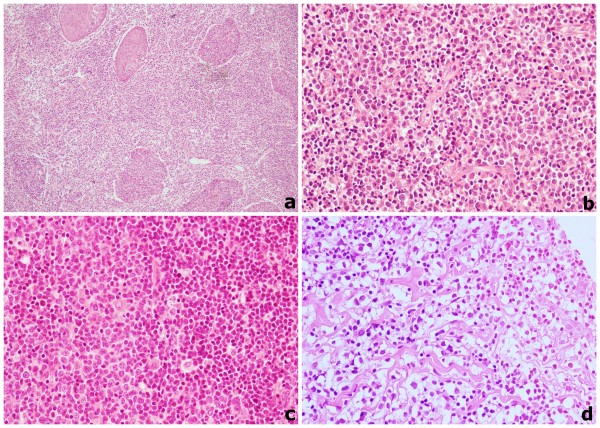
**(a) OAML infiltration of the lacrimal gland, showing squamous metaplasia (H&E) (10×); (b) prevalence of plasmocytoid cells in OAML (H&E) (40×); (c) prevalence of centrocytic cells around a residual germinal center in OAML (H&E) (40×); (d) prevalence of monocytoid cells in OAML (H&E) (40×)**.

OAML derives from acquired MALT in orbital region and from probably native MALT in conjunctive. MALT acquisition is consequent to chronic inflammation due to autoimmune disorders or infection [41]. Microscopically neoplastic cells are monocytoid, centrocytic-like or lymphoplasmocitoid (Figure 1). OAML are characterized by prevalence of one of mentioned cell types [30]. In the regional district containing epithelium such as conjunctive and lacrimal gland, lymphoepithelial lesions, i.e. lymphoma cells invasion of epithelial structures, could be observed. Follicular colonization with lymphoma cells invasion of germinal centers is sometimes present. Single or clustered large cells can be also observed. OAML is always associated to non-neoplastic cells and reactive secondary follicles.

**Figure 2:**
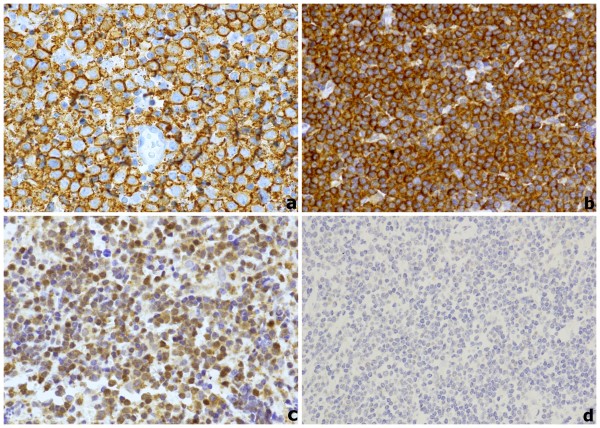
**CD20 and Bcl10 immunohistochemical expression; (a) Diffuse CD20 staining (60×); (2) High cytoplasmic Bcl10 expression (40×); (3) High nuclear Bcl10 expression (40×); (d) Negativity for Bcl10 (40×)**.

Immunoprofile is similar to other MALT lymphoma, being neoplastic cells CD20+ (Figure 2a), CD79a+, CD3-, CD5-, CD10-, bcl-6-, IgM+, bcl-2+, CD43+/-, CD23-/+, CD21+/-, TCL1+, PAX5+, CD11c+/-, CD35+/-, and IgD-, cyclin D1-, MUM1- [5,30]. Differential diagnosis of OAML is generally with other small cells lymphomas, such as mantle cell lymphoma, follicular lymphoma and small lymphocytic lymphoma and with reactive lymphoproliferative disorders. Specific immunohistochemical profile of other low grade lymphomas could help in the correct diagnosis of OAML, while pseudolymphomatous proliferation often requires molecular deepening, taking into account that pathological entities previously classified as 'pseudolymphomas' or 'benign lymphoid hyperplasia' are actually considered very rare, since most of them contain clonal B cells [42].

## Pathogenesis: General mechanisms and infectious agents

**Figure 3:**
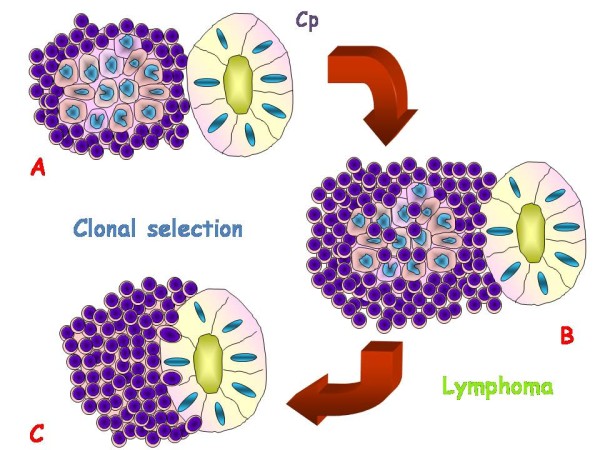
**Schematic representation of lymphomagenesis potentially induced by Chlamydia psittaci (Cp) in acquired MALT with follicular germinal center (A) with consequent clonal selection, characterized by clonal expansion of marginal zone lymphoid cells invading (B) and then replacing germinal center (C)**.

**Figure 4:**
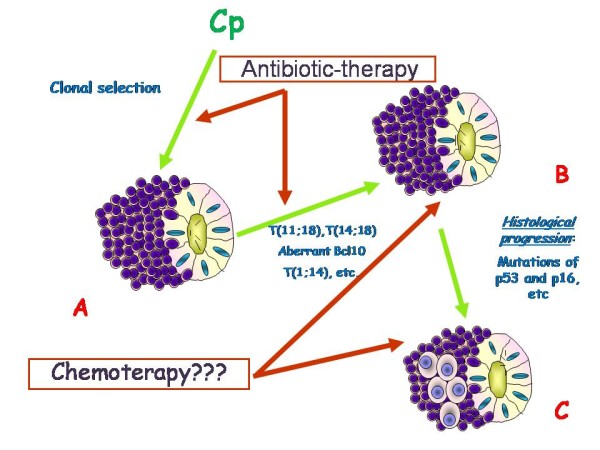
**Schematic representation of OAML biological progression from Cp-clonal selection (A) to lymphoma with chromosomal aberration (B) and finally to histological progression to Diffuse Large B cell Lymphomas (C)**. Antibiotic therapy could be efficacy only in the phase of CP antigen dependent growth.

The MALT lymphomas arise in tissues with native or acquired MALT. When acquired, it derives from reactive lymphoid tissue developed to persistent antigenic stimulation, such as chronic inflammation or an autoimmune disorder. The nature of inflammatory processes in these patients is almost always unknown. As Helicobacter pylori (Hp) plays a relevant role in development of gastric MALT lymphomas, other infectious agents have been investigated in other districts usually sites of MALT lymphomas. Thus for cutaneous B cell lymphomas Borrelia burgodoferi has been found associated in a significant subset, especially in specific geografic areas [10,43-45], while at small intestine Campylobacter jejuni seems to be responsible of MALT lymphoma development [9]. Finally for OAML a similar pathogenic role for Chlamydia psittaci has been proposed. In this context, a B-cell clonal expansion and proliferation could occur (Figure 3), as indicated by the presence of somatically hypermutated immunoglobulin genes with an ongoing mutations pattern. Chronic antigenic stimulation may induce genetic instability with subsequent chromosomal abnormalities, which, associated with the microenvironment, can make the process of clonal growth independent from antigenic stimulation. In addition tumor progression to a more aggressive histologic type of lymphoma (DLBCL) can then be induced by mutations of tumor suppressor genes such as p53 and p16 [46-49] (Figure 4).

The geographical difference in incidence of Cp infection suggests that other agents commonly associated with chronic eye diseases can play a key role in the initiation of neoplasia, as Chlamydia abortus [50], Chlamydia trachomatis, herpes simplex virus type 1 and 2 and adenovirus type 8 and 19 [29,51].

C. pneumonia infection has been hypothesized to be casually associated with different types of malignancies, including cutaneus T-cell lymphoma [52,53], and lung cancer [54], but also B cell lymphomas. Infact C. pneumonia DNA has been detected in a Chinese patient with bilateral orbital MALT lymphoma and then associated with Helicobacter pylori (Hp) in a French patient [53,55]. Hp infection has been also detected in one third of a series of Italian OAML patients and in a series of 15 Korean patients with conjunctiva MALT lymphoma [56,57]. However the association between Hp infection and OAML remains controversial [58]. In fact Sjo et al. failed to identify association between Hp infection and 13 analyzed cases of conjunctival MALT lymphoma through immunohistochemistry and nested polymerase chain reaction (PCR) techniques [30,59,60]. Finally there is increasing molecular and epidemiological evidence of the pathogenetic role of Hepatitis C virus in hepatitis C-associated OAML. Two Italian studies showed HCV seropositivity in 13-36% of patients OAML, which seemed to be associated with more aggressive disease than HCV-negative patients [61,62].

### Chlamydia psittaci in OAML

Chlamydia psittaci is an obligate intracellular bacterium causing pulmonary psittacosis in humans and Chlamydiosis in the avians, including pneumonitis, pericarditis, conjunctivitis, air sacculitis, peritonitis and hepatosplenomegaly [63]. The human psittacosis is generally caused by exposure to infected animals. Morphologically Chlamydophila can present in three different forms: elementary body (EB), reticulate body (RB) and intermediate body (IB). The EB is a small spherical body, of about 0.2-0.3 mm in diameter, characterized by highly electron-dense nucleoid, at the periphery and separated from an electron-dense cytoplasm. The EB is the infectious form, which attaches to the target cell and finally enters it. After entering the host cell, the EB takes the shape of RB (0.5-2.0 mm), which is the metabolically active intracellular form. In the transition from one form to another, an intermediate form IB, measuring about 0.3-1.0 mm, could be observed [63]. Using a combination of PCR and immunohistochemistry, Ferreri et al. detected Cp DNA in 87% of OAML cases in a Northern Italy series, establishing an association between infection of Cp and the OAML [11]. Later, the same group of authors provided further evidences that a complete or partial regression was achieved in some cases of ocular adnexal MALT lymphoma, including cases negative for Cp, following anti-chlamidial antibiotic therapy [11,12,20,64]. Similar findings have been reported from Korean studies, since Cp-DNA was detected in 79% of patients [13]. Finally high prevalence of Cp has been observed in Austrian series [14].

A recent study on ocular adnexal MALT lymphoma cases from six geographic areas showed marked variation of the association between infection and OAML among the regions examined, being most frequent in Germany (47%), followed by the USA (35%), and the Netherlands (29%), but relatively low in central Italy (13%), the UK (12%) and southern China (11%) [15]. Low prevalence (10%) of Cp infection was also found in cases from Cuba [16]. Moreover studies from the South Florida and Rochester (New York) areas of the USA, Netherland, Japan, Africa and Northern China, France have reported absence of the association between Cp infection and OAML [17-25]. Another recent study comparing cases of Central Italy and African OAML underlines a low frequency of Cp infection in Italian series, accounting almost 16% of cases, and absence of Cp infection in African cases [17]. All these findings suggest that there is a geographic variability for Cp association and OAML and that other etiological factors may be involved in the development of this lymphoma [17].

Recently the role of Cp has been proposed in the pathogenesis of other non gastrointestinal MALT, since Cp DNA has been recently evidenced as in all tested lung MALT lymphomas, in 30% of thyroid MALT lymphomas, 25% of skin MALT lymphomas and 13% of salivary gland MALT lymphomas. Moreover Cp has been revealed in 41% of the autoimmune disease Sjögren syndrome and Hashimoto thyroiditis, as potential precursor of specific subsets of MALT lymphomas. A potential, direct cancerogenic role of Cp has been suggested. In fact, these microorganisms, which establish persistent infections, are mitogenic *in vitro *[65], cause resistance to apoptosis in infected cells [66] and induce polyclonal cell proliferations *in vivo *[67,68].

Heat-shock proteins produced by Cp may trigger immune responses, both humoral and cell-mediated, which can generate a cross-reactivity against human proteins and other self antigens [69]. This phenomenon may contribute to alter local tolerance, leading to chronic stimulation by antigens [70]. The role of Cp is not only restricted to lymphoid proliferation induction, but it seems to be responsible of chromosomal aberration, probably due to its mitogenic activity or indirectly induced oxidative damage [26,65]. As in gastric MALT lymphomas t (11;18) were significantly associated with CagA-positive strains of H pylori, probably due to oxidative damage on DNA induced by CagA-positive strains of *H pylori*, in OAML could be suggested the same phenomenon [26]. In fact some infectious agents, as *H pylori *strains harboring the CagA island, cause a potent neutrophil activation through interleukin-8 activity [71]. Finally neutrophils activation is responsible of oxygen-reactive release, causing DNA damage, as double-strand breaks [72]. Whatever the mechanism responsible for chromosomal aberration occurs, the clonal expansion would virtually become independent of antigenic stimulation. Thus frequently infectious agents are not detected in MALT lymphomas with chromosomal aberrations [20]. In fact in gastric MALT lymphomas the presence of t(11;18)(q21;q21) correlates with resistance to antibiotic therapy, since neoplastic proliferation has become independent from infectious disease [73-75]. In addition, relative unfrequent positivity of Cp DNA in diffuse large B-cell lymphomas respect to OAML, is probably consistent with the possibility that the Cp can lead marginal zone lymphomas to progress to a more aggressive histotype (i.e., diffuse large B-cell lymphomas), which is no longer responsive to (and dependent on) the antigenic stimulation provided by the microorganism [11].

### Genetic aberration

Recurrent genetic abnormalities have been described in extranodal MALT lymphomas. Trisomy of chromosome 3 and chromosome 18 are observed in up to 68% and 57% of patients, respectively [27,57,76-78]. Gain of chromosome 3 has been shown to be more common in orbital, than in lacrimal gland and conjunctival OAML [76]. Trisomy of chromosome 18, instead, is more frequent in the conjunctival OAML and predominantly affects young women [78]. Comparative genomic hybridization (CGH) carried out in 10 OAML cases showed recurrent chromosomal gains at 6p21 and 9q33-qter, in addition to trisomy 3, 12 and 18 [79].

**Figure 5:**
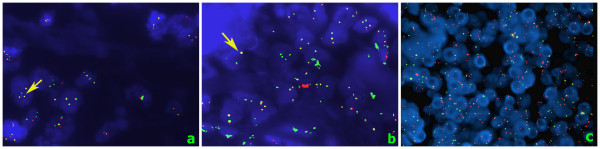
**FISH; (a) T (11;18) (100×)**. Yellow fusion signals (arrow) of API2 (green) and MALT1 (red) translocation; (b) T (14;18) (100×). Yellow fusion signals (arrow) of IGH (green) and MALT1 (red); **(c) **T (1;14). (60×) Dual color break Apart rearrangement BCL10 probe. Following the translocation, there is a split signal.

One of the most frequent translocation in MALT lymphoma is t (11;18) (q21;q21) (Figure 5a). It is demonstrated in 10-50% of gastric MALT lymphomas, whereas this translocation rarely occurs in non-gastric MALT lymphomas, with the exception of pulmonary MALT lymphomas [27]. Translocation fuses the N-terminal region of the API2 (apoptosis inhibitor 2) gene (located at chromosome 11) to the C-terminal region of the MALT1 gene (located at chromosome 18), creating the API2-MALT1 chimeric fusion protein [80-83], which gains the ability to activate the NF-kB pathway [84,85]. However the most common translocation in ocular adnexal MALT lymphomas is the t(14;18) (q32;q21) (Figure 5b), which seems to be one of the main pathogenic mechanisms leading to reduced apoptotic activity, resulting in uncontrolled expression of MALT1 by bringing the *MALT1 *gene under the control of the *IGH *enhancer, a mechanism similar to that used by t(1;14). Also t(1;14) (p22;q32) (Figure 5c) does not form a fusion gene, but rather brings the entire coding region of the *BCL10 *gene on chromosome 1 under the control of the enhancer region of *IGH *gene on chromosome 14, leading to uncontrolled expression of the *BCL10 *gene [86]. Bcl10 is an intracellular protein that is essential for both the development and function of mature B and T cells. Recent studies show that BCL10 specifically links antigen receptor signaling in B and T cells to NF-kB activation [87,88]. In MALT lymphomas arising at gastrointestinal locations, it is claimed that MALT1, with or without bcl10 cooperation, activates the phosphorylation cascade, leading to IkB-α phosphorylation. IkB-α is a physiological ligand of NF-kB in the cytoplasm, whose phosphorylation permits the liberation and migration of NF-kB into the nucleus, where NF-kB plays its transcriptional role, up-regulating the expression of cell cycle regulators, anti-apoptotic proteins, growth factors, negative regulators of the NF-kB pathway and immunoregolatory cytokines [89-94].

Initially immunohistochemical evaluation of Bcl10 has been proposed as a good surrogate marker of translocations in MALT lymphomas. In fact the nuclear expression of bcl10 could suggest NF-kB activation after t(11;18)(q21;q21), t(1;14)(p22;q32), while strong cytoplasmic perinuclear expression seems to be linked to t(14;18)(q32;q21) [28,89,95-97]. It was later noticed that a certain percentage of OAML missing t (11;18) [26] or t (1;14) [29] and lymphoplasmacytoid lymphomas [98] which lack a specific Bcl10 translocation showed a moderate nuclear Bcl10 expression. Moreover in another study an association between weak cytoplasmic Bcl10 expression and translocation (14;18) has been found in only 3 cases [5].

Recently, FOXP1 (located at 3p14) was identified as a new translocation partner of IGH (q32) at low frequency in MALT lymphomas and in DLBCL [99,100].

In another recent study of 29 extragastric MALT lymphomas, the cases of ocular adnexal MALT lymphoma showed the ODZ2-IGH (5;14), JMJD2C-IGH (9;14) new translocations and two translocations with unknown partners of IGH [101,102]. In a case of conjunctival MALT lymphoma has been reported yet another new translocation, the t(5;11) (q33;p11.2), not previously described [103].

Recently, another possible mechanism for uncontrolled NF-kB activation in MALT lymphoma, particularly in the ocular adnexa, salivary gland and thyroid MALT lymphoma, is generated by homozygous deletion of the chromosomal band 6q23 with subsequent loss of the tumor necrosis factor alpha-induced protein 3 (TNFAIP3, A20) [104], an essential global NF-kB inhibitor. A20 is also inactivated frequently by somatic mutations [105-107]. In OAML, A20 inactivation is associated with poor lymphoma-free survival [104,105,108,109] and with a range of chronic inflammatory disorders [110-114].

## Treatment

The treatment depends upon the variables related to the patient (age, autoimmune disorders) and to the disease, particularly on site, stage and surgical accessibility. Surgical excision is most appropriate for many stage 1 conjunctival and lachrymal gland MALT lymphomas, especially in pseudoencapsulated lesions [30]. 'Wait and watch' strategy after surgical resection in patients with stage I disease produces similar results to those reported with immediate radiotherapy, in terms of progression, systemic dissemination, transformation in an advanced stage disease and death associated with lymphoma, with a 10-year overall survival of 94% [38]. The localized low grade OAML can be successfully managed with local radiotherapy. Ocular complications of radiotherapy, including cataract, retinal disorder, xerophthalmia and glaucoma, are frequently reported in patients that have long-term follow up [115]. There are few data in the literature regarding the use of chemotherapy in patients with OAML. Is it clear that is more effective administration of individual chemotherapeutic agents, as chlorambucil or fludarabine, for low-grade lymphomas, and the combinations of chemotherapy, as cyclophosphamide, doxorubicin, prednisone and vincristine, for high grade lymphomas [39,116]. The largest experience regards chlorambucil, an alkylating agent largely used in indolent lymphomas. This drug is an active and well-tolerated therapy for stage I OAML, with a 67-100% response rate, a 79% complete remission rate, recurrence in up to 29% and a 5-year relapse-free survival of 60% [30,97,112]. Immunotherapy includes interferon α and rituximab (anti-CD20 antibody). Rituximab is a monoclonal antibody against CD20 positive B cells, which is being considered as an alternative first line treatment for localized CD20 positive OAML to avoid the ocular complications of radiotherapy [117-120]. As for the gastric MALT lymphoma, antibiotic therapy, aimed to eradicate the Hp infection, is followed by lymphoma regression in 60-70% of stage IE cases [121]. Thus also for OAML associated with Cp infection an antibiotic therapy has been proposed. Results following first line antibiotic treatment are variable [122]. Ferreri et al. documented an encouraging response following oral doxycycline for 3 weeks in 4 out of 9 patients with Cp positive OAML [12]. Abramson et al. showed a clinical response following antibiotic treatment in 3 patients with OAML [123], but Grunberger et al. did not obtain the same results. In another study 27 patients with OAML were treated with oral doxycycline [124]. Cp infection positive cases had an overall response rate of 64%, and also the Cp DNA negative cases showed a clinical response with a rate of 36%, indicating that doxycycline could be used in most OAML patients, independently of the diagnosis of Cp infection [30,64,117]. In a retrospective analysis of 38 patients with localized OAML, Kim et al. evaluated the effect of doxycycline for 3 weeks (12 patients) or 6 weeks (26 patients) [125]. After a median follow-up of 26.4 months, doxycycline resulted in an overall response rate of 47%. Patients treated with doxycycline for 6 weeks versus 3 weeks tended to have a higher response rate (54% vs 33%).

Doxycycline may be also a valid therapeutic alternative in disseminated OAML [126]. 6 patients with disseminated Cp-positive OAML were treated with doxycycline. After treatment, Cp DNA was not detectable in peripheral blood mononuclear cells of the six patients, and after a median follow-up of 31 months, three patients achieved an objective response [126].

## Conclusions

The diagnosis and therapy of OAML requires a strict multidisciplinary approach. In this context the presence of Cp and the occurrence of specific chromosomal aberrations could add important information in order to better personalize the therapy. However further insights are needed about Cp induced lymphomagenesis, in particular with regard to specific antigens related to lymphoma occurrence and the role of the host immunity in lymphoma progression. Finally the absence of Cp in most patients with OAML, even in front of a therapeutic response to antibiotic-therapy, induces on the one hand to improve the diagnostic tool of Cp infection on biological samples from OAML patients and on the other to seek further infectious agents potentially responsible of tumor initiation.

## Competing interests

All authors of this manuscript report no competing interests with respect to any financial or personal relationships with other people or organizations that could inappropriately influence our work.

## Authors' contributions

RF and FC have dealt with the drafting of the manuscript. ADC, GB, GDR, ADR and RF have made a critical revision of the manuscript by introducing important intellectual content. All authors read and approved the final approval version of the manuscript.
